# Facial expression analysis and machine learning for objective assessment of alcohol craving in alcohol use disorder: a study protocol

**DOI:** 10.3389/fpsyt.2025.1693193

**Published:** 2025-11-11

**Authors:** Lanci Liu, Zixiu He, Yuting Song, Guiming Zuo, Xue Bai, Hanshuo Su, Dan Wang, Haoyu Zhao, Shilin Wang, Wenhui Li, Chuansheng Wang

**Affiliations:** 1 The Second Affiliated Hospital of Xinxiang Medical University, Henan, XinXiang, China; 2 Henan Collaborative Innovation Center of Prevention and Treatment of Mental Disorder, Henan, XinXiang, China; 3 Brain Institute, Henan Academy of Innovations in Medical Science, Henan, XinXiang, China

**Keywords:** alcohol use disorder, facial expression analysis, machine learning, facial action coding system, alcohol craving

## Abstract

**Background:**

Alcohol craving is a key predictor of relapse in alcohol use disorder (AUD), yet current assessments rely mainly on self-reported scales, lacking objective evaluation methods. This single-center observational study aims to explore subtle differences in facial expressions among alcohol use disorder patients during craving states, long-term abstinent individuals, and healthy controls, with the goal of identifying objective biomarkers of alcohol craving. A secondary aim is to establish a rigorous craving evaluation system using machine learning.

**Methods:**

We plan to recruit 200 participants per group: alcohol use disorder patients, long-term abstinent individuals, and healthy controls. Patients with alcohol use disorder will first undergo inpatient detoxification (approximately two weeks) and will be eligible once their Clinical Institute Withdrawal Assessment for Alcohol, Revised (CIWA-AR) score falls below 7. At enrollment, participants will complete psychological and clinical assessments, including sociodemographic and drinking history questionnaires, the Alcohol Use Disorders Identification Test (AUDIT), Penn Alcohol Craving Scale (PACS), Hamilton Anxiety Rating Scale (HAM-A), Hamilton Depression Rating Scale (HAM-D), Barratt Impulsiveness Scale, and Pittsburgh Sleep Quality Index (PSQI). Personalized drinking-environment preferences will be collected via semi-structured interviews. During the experiment, participants will provide craving ratings using a visual analog scale (VAS) before and after viewing a 120-second relaxation video and a 120-second alcohol cue-related video, while facial expressions are recorded simultaneously. The alcohol use disorder group will undergo the same assessments again after six weeks of inpatient treatment.

**Discussion:**

Developing an objective system for evaluating alcohol craving has the potential to enhance routine screening, differential diagnosis, and treatment monitoring in alcohol use disorder. Furthermore, integrating facial expression analysis with machine learning may enable the development of reliable craving assessment tools and treatment response prediction models. Such approaches could provide clinicians with evidence-based guidance for psychological and psychosocial interventions, ultimately reducing relapse rates among individuals with alcohol use disorder.

## 1 Introduction

Alcohol use is a major global health issue, contributing to approximately 3 million deaths annually and accounting for 5.1% of global disability-adjusted life years (DALYs). Studies indicate that alcohol consumption significantly increases the risk of more than 60 diseases in Chinese men (1).

### 1.1 Alcohol craving and cue reactivity: mechanisms and assessment challenges

Alcohol Use Disorder (AUD) is a chronic, relapsing brain disease in which craving plays a pivotal role in driving relapse after abstinence (2, 3). Evidence suggests that the intensity of craving in early treatment reliably predicts subsequent heavy drinking and relapse risk, reflecting its close association with impaired self-control (4). Thus, accurate monitoring and management of alcohol craving are critical to improving alcohol use disorder treatment outcomes.

Alcohol-related cues, such as odors, images, or drinking environments, further exacerbate craving by activating neural circuits including the Dorsal Anterior Cingulate Cortex, Nucleus Accumbens, Posterior Cingulate Cortex, Pregenual Anterior Cingulate Cortex and orbitofrontal cortex (5–7), a phenomenon also consistently observed in psychological assessments (8).

Yet, despite its clinical importance, craving remains difficult to evaluate: Due to their self-reported nature, subjective biases in self-report scales cannot be completely avoided. These biases not only affect the accuracy of assessment results but may also further interfere with clinical diagnosis, the formulation of treatment plans, and the monitoring of treatment efficacy. Taking the Penn Alcohol Craving Scale (PACS) as an example: patients may exhibit biases in self-reports under the influence of factors such as social desirability (9), cognition (10), memory, situational dependence, and response set (11). Participants may deliberately conceal or downplay their actual craving levels due to reasons such as unwillingness to be labeled as addicted (12) or a desire to conform to social expectations of healthy behavior (13); alcohol craving is a subjective psychological experience, and differences in how individual participants perceive this concept lead to inconsistent scoring standards; some items of the PACS rely on participants’ short-term memory, yet alcohol-dependent individuals may have memory biases due to cognitive impairment (14) caused by long-term alcohol consumption; participants’ scores may also be affected by the physical environment (15), emotional state, or interpersonal interactions during the assessment, rather than fully reflecting their true craving levels. Therefore, although the measurement results of subjective reports are of great reference value, it is still necessary to explore more comprehensive objective evaluation indicators as much as possible.

While objective approaches (e.g., Eye-Tracking, Skin Conductance Response/Heart Rate Variability, Ecological Momentary Assessment) combined with machine learning (16, 17) remain fragmented, they have shown potential in improving the accuracy of psychological assessments. The lack of a standardized, multimodal assessment system not only limits accurate characterization of craving but also constrains the development of targeted interventions. These gaps highlight the urgent need for integrative, multidimensional approaches to better capture craving dynamics and improve treatment outcomes in alcohol use disorder.

### 1.2 Facial expression analysis: a new perspective in mental illness assessment

Facial expressions, as powerful and universal indicators of emotional states, play a significant role in diagnosing diseases and accurately assessing inner states (18). The Facial Action Coding System (FACS), developed by Paul Ekman and Wallace Friesen in 1976, is a standardized, anatomy-based analytical tool. Its core lies in decomposing facial muscle movements into 44 independent Action Units (AUs). By quantifying the activation intensity, duration, and combination patterns of these units, FACS enables the objective interpretation of human emotional expressions. It overcomes the limitations of traditional subjective emotional judgment by breaking down subtle facial muscle movements (i.e., Action Units) to achieve precise description and interpretation of expressions (19). Currently, FACS is widely applied in fields such as psychology, neuroscience, human-computer interaction, and forensic investigation.

Facial Action Units (AUs) are the minimal analytical units of the FACS. They refer to specific observable facial movements produced by the coordinated contraction or relaxation of one or more facial muscles (e.g., frowning, eye opening, lip corner elevation). Importantly, AUs only describe observable muscle movements and are not directly associated with specific emotions. A single AU can appear independently (e.g., eye blinking alone, corresponding to AU45) or combine with other AUs to form complex expressions (e.g., smiling is often composed of the combination of AU6 and AU12). Each AU corresponds to a specific group of facial muscles, for instance, AU1 refers to the elevation of the inner part of the eyebrow, AU2 to the elevation of the outer part of the eyebrow, AU5 to the raising of the upper eyelid, and AU14 to the contraction of the corners of the mouth towards the teeth (20).

Through extensive cross-cultural studies (including research on isolated indigenous tribes), Ekman confirmed that humans exhibit 6 basic emotions (happiness, sadness, anger, fear, surprise, and disgust). Each basic emotion corresponds to a set of characteristic AU combinations—that is, the coordinated activation of specific AUs forms universally recognizable emotional expressions (21). For instance, happiness is indicated by AU6 + AU12, while pain is represented by AU4 + AU6/7 + AU25 (22). FACS’s high sensitivity makes it particularly effective for detecting subtle emotional signals during cue exposure.

FACS has wide applications in mental health research. It has been used to identify emotional expression deficits in schizophrenia (e.g., insufficient activation of AU12) (23), examine Post-Traumatic Stress Disorder patients’ abnormal responses to threatening stimuli (24) (e.g., frequent activation of AU4), and improve disease diagnosis and prediction using AI-driven systems (23) (e.g., predicting schizophrenia recurrence). FACS is now integrated with technologies such as eye-tracking, voice analysis, and functional Magnetic Resonance Imaging to create multi-dimensional emotional analysis platforms (25).

### 1.3 Facial expression characteristics of alcohol use disorder patients: exploration and gaps

Research on facial expressions in patients with alcohol use disorder is currently in the exploratory stage. At present, there is no direct research analyzing the characteristics of facial expressions in patients with alcohol use disorder during alcohol craving states. Existing direct evidence mainly focuses on impairments in emotion recognition ability (26); there are also studies that have indirectly inferred that patients with alcohol use disorder may exhibit abnormalities in the dynamic expression of facial expressions, and such abnormalities are mostly closely associated with neurocognitive dysfunction and abstinence status.

Potential dynamic abnormalities in facial expression production: patients with alcohol use disorder (even those who have achieved abstinence) exhibit significantly reduced brain activity in the amygdala and hippocampus (27) when processing others’ facial expressions. These two brain regions are closely associated with emotional perception and memory; impairments in their function may lead to biases in patients’ judgment of emotional intensity, which in turn affects the production of their own facial expressions. Event-Related Potentials studies on hazardous drinkers (HDS) have shown delays in the early emotional processing stage (e.g., P1 and N170 components) (28), suggesting that the generation of facial expressions may appear dull or lack variability due to slowed neural conduction velocity. Patients with alcohol use disorder exhibit not only impairments in facial expression recognition but also similar deficits in the decoding of emotional prosody (e.g., emotional speech) and body postures (29). This suggests that a decline in overall emotional processing ability may affect the diversity of their own facial expressions.

The relationship between these abnormalities and facial action unit (AU) activity, particularly during alcohol cue-induced craving, remains underexplored.

### 1.4 Abstainers (previously diagnosed with alcohol use disorder, now abstinent)

In existing literature, there are relatively few studies directly targeting the facial expressions of alcohol abstainers, and the differential characteristics of facial expressions under alcohol cues have not yet been clarified. Most existing studies focus on neural mechanisms and certain physiological changes.

Under alcohol cue induction, abstainers exhibit abnormal activation in the anterior cingulate cortex (ACC) and dorsolateral prefrontal cortex (DLPFC) (30). In alcohol-related Go/No-Go tasks, individuals with high craving show more significant topological differences in the N2 component (which reflects inhibitory conflict), while the P3 component can distinguish relapsers from sustained abstainers (31).Cue-induced craving is associated with emotions. Alcohol-related cues (e.g., images, odors) can elicit craving in abstainers, accompanied by increased heart rate and changes in cortisol levels (32, 33),however, no systematic assessment of facial expressions has been observed.

In summary, while abstainers exhibit distinct neural, physiological, and behavioral characteristics, research on facial expressions—particularly as indicators of craving—is lacking. This gap presents an important opportunity to use technologies like FACS combined with alcohol cue tasks to explore expression differences and the objective biomarkers of craving states.

### 1.5 Insights from related studies (depression and addictive diseases)

At present, research on facial expressions in patients with depression is more extensive; therefore, the relevant studies on depression are briefly summarized here. Depression: Clinically cured individuals show gradient differences in facial expression recognition, AU activity, and neural mechanisms compared to patients and healthy individuals. This gradient feature offers diagnostic, assessment, and relapse warning potential (34–36).

Researchers have analyzed the facial expression characteristics of smokers when exposed to cigarette cues (37) and people with a drug use disorder when exposed to cocaine cues (38). Experimental results have shown that facial expression features exhibit differential changes during states of craving. However, studies exploring differences in facial expression features among active addicts, long-term abstainers, and healthy individuals under the induction of relevant cues remain relatively scarce. The following are some relevant studies:

Addictive Diseases: Studies on other addictions (e.g., nicotine and drug use disorder) reveal contradictory micro-expressions and dynamic facial responses under cue exposure. For example: People with a nicotine use disorder: Display contradictory micro-expressions (e.g., AU12 for pleasure and AU4 for negative emotions), reflecting the tension between craving and cessation. Facial Electromyography (EMG) recordings demonstrate that withdrawal can enhance the expression of negative emotions (37, 39–41). Smoking Quitters: Exhibit higher rates of contradictory micro-expressions when exposed to cues, correlating with relapse risk (40, 42–44).

Drug Abstainers: Show impairments in basic emotional processing, such as difficulty recognizing fear/sadness (45, 46). Abstinent individuals appear to exhibit no difference from active drug users in terms of implicit responses when exposed to drug cues, whereas differences seem to exist in their explicit characteristics (47). The nucleus accumbens function in opioid abstainers shows partial recovery compared to that in active opioid addicts, yet the recovery remains incomplete relative to healthy individuals (48).

These studies provide crucial theoretical and methodological insights into exploring facial expressions, particularly contradictory micro-expressions and dynamic AU patterns, to assess alcohol craving and predict relapse risk.

### 1.6 Machine learning: a new tool for craving assessment

Machine learning (ML) excels at integrating multimodal data (e.g., AUs, physiological signals) to build diagnostic, assessment, and prediction models (49). To address the aforementioned subjective limitations of self-reported psychiatric scales, constructing diagnostic assessment models based on unimodal or multimodal objective indicators and machine learning algorithms has become a core approach to overcoming the bottlenecks of traditional assessment methods (50). Guided by the core logic of objective indicator quantification - feature engineering optimization - algorithm modeling and validation, this method converts psychopathological states into computable high-dimensional feature vectors through facial micro-expression features (encoded based on Facial Action Units, AUs), thereby enabling accurate classification and severity assessment of psychiatric disorders (51). However, applying ML to facial expression analysis in alcohol use disorder individuals during craving states is an emerging area, vital for achieving an objective and dynamic assessment system.

### 1.7 Research objectives

This study aims to use facial expression analysis (combined with machine learning) as an objective indicator to assess alcohol craving. The research objectives are:

1. Reveal Craving Expression Characteristics: Identify subtle differences in facial expressions (especially micro-expressions and AU activities) between alcohol use disorder individuals in craving and relaxed states, providing an objective basis for craving assessment.
2. Identify Key Groups: Compare the facial expression characteristics of long-term abstainers in craving states with those of non-abstainers and healthy controls to identify biomarkers of craving, while facilitating better clinical screening and differentiation of the three populations.
3. Build an Intelligent Recognition Model: Develop an automated model to recognize alcohol craving states based on differential facial expression characteristics using machine learning.
4. Evaluate Efficacy: Use the model to assess changes in alcohol craving before and after conventional inpatient treatment and explore constructing a prediction model for treatment efficacy based on baseline expression characteristics.
5. Prediction of relapse risk: Through a 6-month follow-up, this study aims to verify the predictive value of facial action unit features at the baseline period for the risk of relapse in patients with alcohol use disorder (AUD) after treatment, and to explore the effectiveness of these features as a relapse prediction indicator. Additionally, it intends to evaluate the association between facial action unit features in AUD patients after 6 weeks of treatment and their relapse outcomes at 6 months.

### 1.8 Research hypothesis

Based on the aforementioned current state of research, relevant studies, and the objectives of this study, we propose the following hypothesis: The potential differences among groups (similar to the Nicotine Addicts in Section 1.5) include: Compared to healthy individuals: Healthy people have weak responses to alcohol cues, strong regulation, and may express neutral emotions (e.g., AU0). Compared to non-abstainers: Non-abstainers may show stronger craving and impulsivity, potentially exhibiting approach-related expressions (e.g., AU12 for pleasure). Among abstainers themselves: Abstainers may display contradictory expressions (e.g., initial approach followed by inhibition), which requires empirical verification.

## 2 Methods and analysis

### 2.1 Study design and setting

This study is a mixed design, incorporating both cross-sectional and longitudinal components, on adult male patients with alcohol use disorder, with an estimated total research duration of two years (from August 2025 to June 2027). The proposed study site for subject recruitment is the Second Affiliated Hospital of Xinxiang Medical University, Henan, China.

### 2.2 Groups and sample size

This study will be divided into three groups: individuals with alcohol use disorder, long-term abstainers, and healthy controls. A total of 200 subjects are expected to be recruited for each group to participate in the experiment, and the information collected from each subject will be categorized by group.

To ensure sufficient statistical power to detect the primary outcome (i.e., differences in facial AU activities across the three groups), we calculated the sample size following standard power analysis procedures (α = 0.05, two-tailed; statistical power = 0.80). The estimation was based on a one-way ANOVA across three groups. We set the partial eta-squared (η²) to 0.10 (representing a medium-to-large effect size according to Cohen’s conventions). Using the effect size conversion formula f = sqrt (η²/(1 - η²)), the corresponding Cohen’s f was approximately 0.333. Power analysis with G*Power (one-way ANOVA, groups = 3, α = 0.05, power = 0.8, f = 0.333) indicated a required sample size of approximately n = 112 per group. For two-group comparisons (e.g., AUD vs. healthy controls in VAS scores), assuming Cohen’s d = 0.70 (medium-to-large effect size), a minimum of n = 64 per group will be required. Considering multiple analytic scenarios, subgroup analyses (e.g., stratification by medication), and a potential dropout rate of ~20% reported in similar inpatient/follow-up studies, we ultimately decided to enlarge the sample size and adopt a conservative strategy of recruiting 200 participants per group (600 in total). This strategy ensures robust statistical power and reliability across both primary and secondary analyses.

### 2.3 Recruitment of subjects

**Figure 1:**
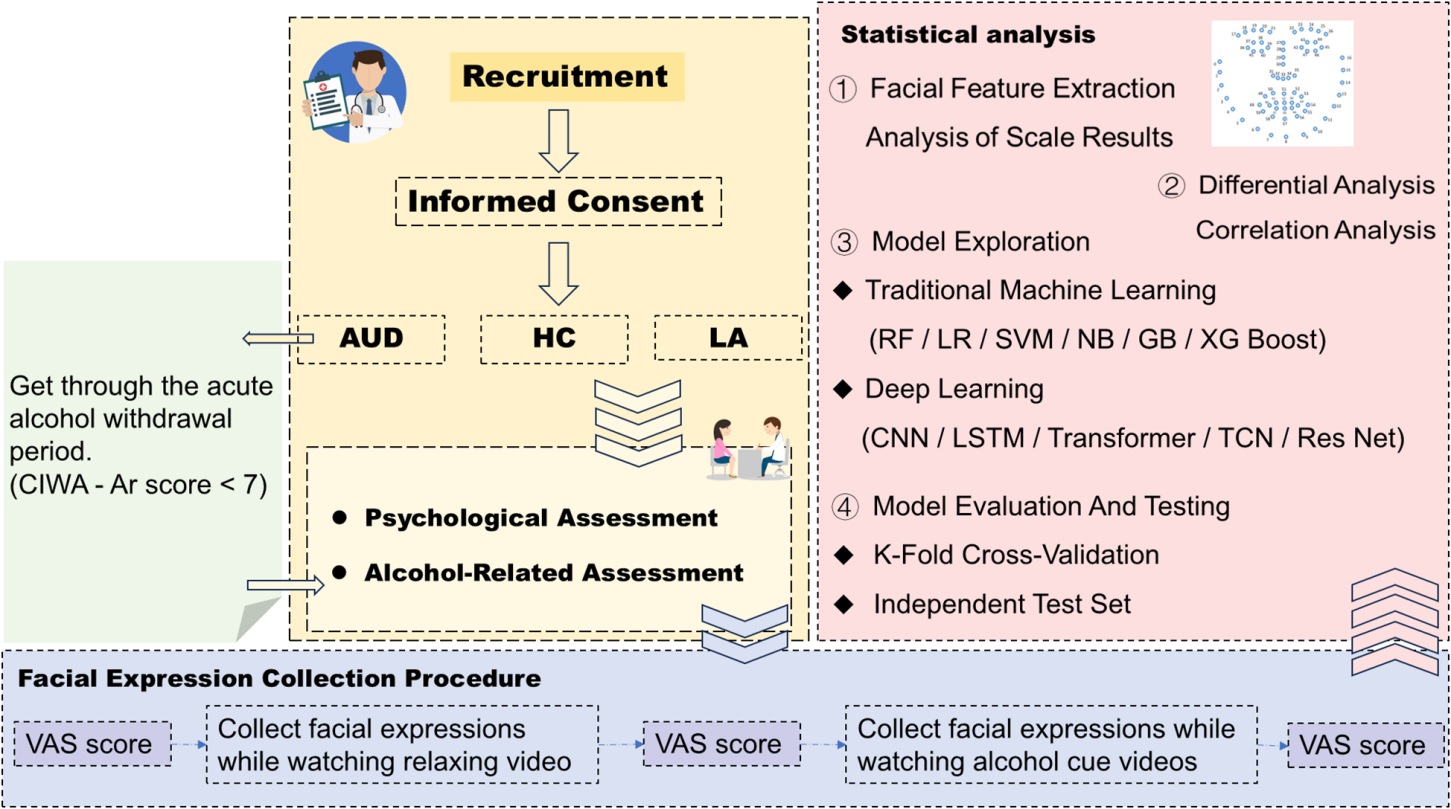
Experimental process of the present research.

**Figure 1** is the complete experimental flowchart. The source population will be comprised of male patients diagnosed with alcohol use disorder who were hospitalized in the Inpatient Department of Addiction Medicine, the Second Affiliated Hospital of Xinxiang Medical University (only male patients are selected as the prevalence of alcohol use disorder is much higher in men than in women and the majority of patients presenting to the Addiction out-patient clinics are men in China (52), which is different from Western culture). The sources of long-term alcohol abstainers will include community-based alcohol support groups (e.g., Alcoholics Anonymous, AA) and patients who previously completed alcohol detoxification treatment at this hospital and remained abstinent without relapse during an 8-10-year follow-up period, while healthy controls will be recruited from community residents and individuals undergoing health check-ups at this hospital. Potential participants will be approached by a research assistant and will be provided with an informational brochure detailing the study objectives, procedures, and duration. Their feedback will be obtained to determine interest in participation. The research assistant will explain the contents of the brochures to the potential patients to ensure that they understand and are clear with the information about the study. Patients who voluntarily agreed to participate in the study will be screened for inclusion and exclusion criteria.

The participants will be divided into three groups in total: AUD group: Hospitalized patients who meet the DSM-5 diagnostic criteria for alcohol use disorder. Long-term Abstainers group: Individuals with a confirmed history of alcohol use disorder (AUD) who were discharged from abstinence treatment, and have maintained either no alcohol consumption or only moderate drinking over the past 8–10 years without meeting the diagnostic criteria for heavy drinking or recurrent AUD. Their general conditions are matched with those of the alcohol use disorder group. Healthy Controls group: Healthy individuals who meet the standard of moderate alcohol consumption specified in Chinese Dietary Guidelines (with daily pure alcohol intake not exceeding 15g), and their general conditions are matched with those of the alcohol use disorder group. Specific Exclusion Criteria: individuals whose alcohol consumption exceeds the light drinking threshold (53) (i.e., those with weekly alcohol intake of more than 7 standard drinks) shall be excluded.

The inclusion criteria are as follows (applicable to all groups): 1.Male, aged between 18 and 55 years;2.Having an educational level of junior high school or above;3.No obvious physical withdrawal symptoms (Clinical Institute Withdrawal Assessment for Alcohol score < 7);4.All subjects are right-handed;5.Normal vision or corrected vision, without color blindness or color weakness, and without neurological diseases or facial-related diseases;6.Able to cooperate with the examination;7.Having obtained the informed consent of the patients;8.Able to provide accurate information on alcohol consumption.

The exclusion criteria are as follows (applicable to all groups):1.Having a history of severe physical diseases or organic brain diseases;2.Having a history of substance abuse other than alcohol and nicotine;3.Having a history of other mental illnesses;4.Having auditory or visual problems that hinder video viewing;5.Facial paralysis;6.Being unable to cooperate or refusing to complete relevant treatments and examinations;7.Having a history of facial surgery or cosmetic interventions that affect facial expressions;8.Having used any prescription or over-the-counter drugs that may affect mood or alcohol craving within the past 30 days.

Eligible participants will be invited to join the study and, prior to providing written informed consent, will be informed of the study objectives, procedures, potential risks and benefits, withdrawal rights, data anonymity and storage, and available incentives. Consent will be obtained by a research assistant independent of the study. Recruitment will be facilitated through advertisements posted at the Second Affiliated Hospital of Xinxiang Medical University, and participants will receive an honorarium to cover time and travel expenses.

### 2.4 Measures

In accordance with the criteria of DSM-5, patients with alcohol use disorder are diagnosed by experienced psychiatrists based on structured clinical interviews (the MINI International Neuropsychiatric Interview (Chinese version)) combined with assessments of medical/substance use history, neuropsychological test results, and neurological examinations.

The recruitment process of AUD group will be initiated during the patients’ acute detoxification treatment (i.e., after admission but before their CIWA-Ar scores drop below 7). The detoxification protocol primarily consists of benzodiazepine substitution therapy, adequate supplementation of B vitamins, and, as clinically indicated, the administration of antipsychotics, antidepressants, and other symptomatic supportive treatments. Benzodiazepines will be tapered and discontinued once withdrawal symptoms subside (Clinical Institute Withdrawal Assessment for Alcohol, CIWA-Ar score <7). Patients with AUD will be eligible for study enrollment once they meet the inclusion and exclusion criteria, voluntarily sign the informed consent form, and wait for their CIWA-Ar score to drop below 7, whereas this process is not required for healthy controls and long-term abstinent individuals.

After enrollment, before facial expression collection, the subjects will undergo psychological assessment. The psychological assessment includes the administration of questionnaires on sociodemographic, clinical, and alcohol use characteristics(including past alcohol abstinence history and past medication use history), the Alcohol Use Disorders Identification Test (AUDIT), the Penn Alcohol Craving Scale (PACS), the Hamilton Anxiety Rating Scale (HAM-A), the Hamilton Depression Rating Scale (HAM-D), the Barratt Impulsiveness Scale, and the Pittsburgh Sleep Quality Index to evaluate the subjects’ psychological and living conditions in the recent period. At the same time, a semi-structured interview will be used to assess and collect the subjects’ personal preferences for drinking environments when they usually drink, so as to select the drinking environment that the subjects prefer when choosing alcohol cue-related videos.

Previous studies have shown that spontaneous facial expressions can convey an individual’s emotions without conscious effort (54, 55). In the facial expression task, each subject will be invited alone to a soundproof room with moderate lighting and temperature for facial expression collection. Environmental lighting will be calibrated before facial data collection for each participant. Cool white LED panel lights (with a color temperature of 5500–6500 K, close to natural light) will be used, and LED lights with a Color Rendering Index (CRI) ≥ 90 will be selected to ensure the realistic reproduction of facial details (such as wrinkles and pores). Before each experiment, participants will be asked to face the camera, and the camera’s built-in custom white balance function (or calibration via software such as Logitech Capture) will be used to set the white balance based on the participants’ facial skin tone. This step is intended to avoid facial skin tone discoloration (e.g., reddish or bluish tint) caused by fluctuations in the color temperature of the light source, thereby ensuring that facial features of different participants are extracted in a unified color space The subject’s seat is approximately 50–70 cm away from the desktop camera and the video playback screen. Video data will be captured using an Insta360 Link2 camera, which is positioned at a fixed distance of 70 cm from the participant. The recording uses 24-bit RGB color format with a resolution of 1920 × 1080 pixels, 25 frames per second, and RAW format.

Before the official start, the subjects will be informed of the entire experimental process, which will include three visual analog scale ratings and viewing two videos of approximately 120 seconds each, including a relaxation video and an alcohol cue-related video. After 6 weeks of routine inpatient treatment for alcohol use disorder patients, the second collection of the same process (including psychological assessment and facial expression collection) will be conducted for them, which is not required for healthy people and long-term alcohol abstainers. During the collection of facial expressions, simultaneous measurement of several physiological indicators (heart rate variability and skin conductance) will be conducted to explore the consistency between changes in facial expressions and changes in physiological indicators. At 3 and 6 months after the completion of inpatient treatment, follow-up assessments will be conducted for the alcohol use disorder group (including psychological assessments and facial expression collection), while the long-term alcohol abstinence group and the healthy control group will not undergo such follow-up assessments.

The alcohol cue-related video content library contains 4 versions of videos (the library will be continuously expanded in the future to meet the needs of participants and pursue individualized selection), including drinking alone at home (in Mandarin and Henan dialect) and drinking with friends outside (in Mandarin and Henan dialect). Based on Chinese drinking habits, the content selection was conducted through pre-interviews with a large number of potential participants, where their preferred drinking scenarios, types of alcohol consumed, and more commonly used language in daily life were collected. This was aimed at maximizing the realism of drinking scenarios to induce inner craving for alcohol and changes in facial expressions. The video content of drinking alone at home depicts a person secretly taking out alcohol to drink alone when no one is at home, accompanied by two simple side dishes. The video content of drinking with friends shows gathering with friends at a restaurant, eating, drinking and chatting together. The content of the relaxation videos consists of local natural landscape scenes of mountains and waters. The future iterations will include standardized valence/arousal ratings of the video clips. The alcohol cue-related video and the relaxation video have been filmed in advance.

#### 2.4.1 Sociodemographic, clinical, and alcohol use characteristics

Participant demographic and clinical characteristics, including age, sex, ethnicity, education, marital status, religion, occupation, income, smoking history, medical and psychiatric history, and current medications, will be recorded.

Alcohol use history will include age at first use, beverage type, drinking frequency, average daily intake, duration of abstinence, past-week binge drinking (≥5 drinks per occasion for men) and the number of hazardous drinks per day or per week (hazardous drinking is defined as 14 drinks per week or four drinks per drinking day for men) (56).

#### 2.4.2 Primary outcomes

##### 2.4.2.1 Differences in facial expressions induced by alcohol cues

In patients with alcohol use disorder, the craving for alcohol and arousal level significantly increase after viewing alcohol-related pictures (8). Based on the aforementioned relevant studies, it can be indirectly inferred that there are still subtle differences in facial expressions among addicts, long-term abstainers, and healthy individuals when induced by relevant cues. As an objective explicit indicator of emotions and cravings, facial expressions are expected to become an objectively existing biomarker (57).

##### 2.4.2.2 Visual Analog Scale

Visual Analog Scale (VAS) is a measurement tool commonly used to quantify subjective feelings (such as pain, satisfaction, fatigue, etc.). Due to its simplicity in operation and high sensitivity, it is widely applied in fields such as medicine, psychology, and rehabilitation science. Subjects are asked to mark a position on a 10-centimeter horizontal line that corresponds to their level of craving, where the leftmost end “0” indicates no craving and the rightmost end “10” indicates an extremely strong craving. The evaluated individuals are instructed to mark the point on the line that best represents their current state based on their own feelings (the degree of wanting to drink at present). A higher score indicates a stronger corresponding feeling (a higher craving for alcohol). Under alcohol cue induction, the difference in the Visual Analog Scale scores before and after induction represents the arousal of alcohol craving (58).

##### 2.4.2.3 Penn alcohol craving scale

A standardized tool will be used to quantify alcohol craving, which is commonly applied in the clinical assessment and treatment monitoring of Alcohol Use Disorder (AUD) and related research. It is particularly suitable for evaluating short-term craving (e.g., past week). The scale includes five items assessing frequency, intensity, duration, difficulty controlling cravings, and behavioral impact, with higher scores indicating greater craving. The instrument has been translated and validated in the Chinese population, demonstrating excellent internal consistency (Cronbach’s α = 0.97) (56).

##### 2.4.2.4 The definition of relapse

The definition of relapse in this study integrates behavioral criteria and quantitative thresholds (59): Patients with Alcohol Use Disorder (AUD), after completing 6 weeks of inpatient treatment and being discharged, meet any of the following criteria during the 3-month or 6-month follow-up: (1) The reoccurrence of drinking behavior that meets the diagnostic criteria for alcohol use disorder in DSM-5 (e.g., loss of control over alcohol consumption); (2) Pure alcohol intake of ≥25g in a single drinking occasion (in accordance with the Dietary Guidelines for Chinese Residents (2022)); (3) A weekly drinking frequency of ≥3 times for more than 2 consecutive weeks.

#### 2.4.3 Secondary outcomes

##### 2.4.3.1 Alcohol Use Disorder identification test

A standardized screening tool developed by the World Health Organization (WHO) is used to assess alcohol-related risk and the severity of Alcohol Use Disorder (AUD) in adults. It identifies harmful drinking and early signs of alcohol use disorder and is widely applied in clinical, community, and public health settings. Higher scores indicate greater alcohol-related risk or disorder severity (60). The tool was translated and validated in the Chinese alcohol use disorder population, and its internal consistency was acceptable with Cronbach’s α of 0.782 (61).

##### 2.4.3.2 Hamilton Depression and Anxiety Rating Scales

The Hamilton Depression Rating Scale (HAM-D, 17 scored items) and the Hamilton Anxiety Rating Scale (HAM-A, 14 items) will be employed to assess depressive and anxiety symptom severity. For the HAM-D (62), scores of 0–7, 8–16, 17–23, and ≥24 indicate normal, mild, moderate, and severe depression, respectively. For the HAM-A (63), scores of <17, 18–24, 25–30, and >30 indicate mild, mild to moderate, moderate to severe, and severe anxiety, respectively. Both instruments have been translated and validated in the Chinese population, with Cronbach’s α values of 0.714 (HAM-D) and 0.93 (HAM-A) (56).

##### 2.4.3.3 The Barratt Impulsiveness Scale

The Barratt Impulsiveness Scale (BIS) is a widely used self-report instrument designed to assess impulsivity, a personality trait characterized by rapid, unplanned reactions to internal or external stimuli without regard to consequences. The 11th version (BIS-11) consists of 26 items across three subscales: Attentional Impulsiveness (e.g., “I have trouble concentrating”), Motor Impulsiveness (e.g., “I act on the spur of the moment”), and Non-Planning Impulsiveness (e.g., “I do things without thinking”). Items are rated on a 4-point Likert scale (1 = rarely/never, 4 = almost always), with 11 items reverse-scored to minimize response bias. Total scores range from 26 to 104, where higher scores indicate greater impulsivity. The Chinese version of the Barratt Impulsiveness Scale (BIS-11) demonstrates robust internal consistency as measured by Cronbach’s α coefficients, with values consistently exceeding the psychometric standard of 0.70 across diverse Chinese populations (64).

##### 2.4.3.4 The Pittsburgh Sleep Quality Index

The Pittsburgh Sleep Quality Index (PSQI) is a self-administered instrument designed to assess sleep quality and disturbances over a 1-month period. Developed by Buysse (65), the PSQI consists of 19 self-rated items (with 18 items contributing to the total score) and 5 items rated by a bed partner, evaluating seven domains: Sleep Quality, Sleep Latency, Sleep Duration, Sleep Efficiency, Sleep Disturbances, Use of Sleeping Medication, and Daytime Dysfunction. Each domain is scored on a 0–3 scale, with a total score ranging from 0 to 21. A higher score indicates poorer sleep quality, with scores ≥5 generally indicating clinically significant sleep disturbances. The Chinese version of the Pittsburgh Sleep Quality Index (PSQI) demonstrates robust internal consistency across diverse populations, with Cronbach’s α coefficients consistently exceeding the psychometric benchmark of 0.70.

##### 2.4.3.5 Heart Rate Variability and Skin Conductance

Heart Rate Variability (HRV) is a crucial objective indicator for assessing the balance of autonomic nervous system function. In the field of addiction (e.g., research on Alcohol Use Disorder, AUD), changes in HRV are often used to determine the degree of physiological arousal induced by alcohol cues, thereby assisting in the evaluation of autonomic nervous system regulation abnormalities during craving states (66). Skin Conductance (SC)/Skin Conductance Response (SCR) is a classic indicator reflecting emotional arousal and stress responses. In alcohol craving research, monitoring changes in SCR induced by alcohol cues (e.g., drinking videos) enables the objective quantification of the intensity of participants’ physiological responses to alcohol cues, which helps offset the biases associated with subjective scale assessments (67).

### 2.5 Data analysis

All statistical analyses will be conducted using SPSS version 26.0 (IBM Corp., Armonk, NY, USA) and Python 3.9. Missing data will be handled via multiple imputation (for scale scores and physiological indices) or listwise deletion (for participants with >20% missing data or entirely invalid facial recordings). All tests will be two-tailed, with a significance level set at α = 0.05.

Facial expression feature extraction will be performed using the open-source toolkit Open Face 2.0. This toolkit employs validated computer vision algorithms capable of reliably detecting facial landmarks, estimating head pose and gaze direction, and identifying facial Action Units (AUs). Processing will follow standardized protocols: video data will be analyzed at 25 frames per second, and for each frame, the presence of each AU (binary variable: 0 = absent, 1 = present) as well as its intensity (ordinal variable: 1–5, representing minimal to maximal intensity) will be estimated. To derive overall activity indicators for each AU, both the mean intensity and the proportion of presence across all video frames will be calculated.

For Research Objective 1, examining associations between facial AU features (e.g., AU4 mean intensity, AU12 presence proportion) and subjective craving scores: AU features (mean intensity, presence proportion) will be an independent variable, and subjective craving scores (PACS total score, VAS score before/after alcohol cue exposure) will be a dependent variable. Pearson’s correlation (for normally distributed data) or Spearman’s correlation (for non-normally distributed data) will be used. Additionally, for exploring AU feature differences between craving (post-alcohol cue) and relaxed (post-relaxation video) states, Analysis of Covariance (ANCOVA) will be applied to control for potential confounders (age, education level, HAM-D/HAM-A scores, and medication use).

What corresponds to Research Objective 2 is comparing facial AU features, subjective craving scores, and physiological indices (HRV, SC) across the three groups (AUD, LA, HC): Group (AUD, LA, HC; categorical) will be an independent variable, and facial features (AU mean intensity, AU presence proportion), subjective scores (VAS [before/after cue], PACS total score), and physiological indices (HRV [RMSSD, LF/HF ratio], SC [mean level, peak SCR frequency]) will be dependent variables. For normally distributed data with homogeneous variance, One-way ANOVA and Tukey’s HSD *post-hoc* test will be used, and partial η² will be reported as the effect size; for non-normal/heterogeneous variance data, Kruskal–Wallis H test and Dunn’s test with Bonferroni correction will be used, and r = Z/√N will be reported as the effect size. Repeating group comparisons using ANCOVA, with covariates including age, education level, HAM-D score, and (for the AUD group) medication use (0 = no, 1 = yes).

For Research Objective 3, an automated machine learning model will be developed to recognize alcohol craving states based on differential facial expression characteristics. Its specific implementation steps are as follows: The first step involves data splitting and feature engineering: the dataset will be randomly split into a training set (accounting for 70%) and an independent test set (accounting for 30%) to ensure unbiased model evaluation. Input features (predictors) will include facial Action Unit (AU) features (mean intensity, presence proportion), physiological indices (Heart Rate Variability [HRV], Skin Conductance [SC]), and baseline Penn Alcohol Craving Scale (PACS) scores. To reduce model complexity and improve interpretability, feature optimization will be performed via standardization, missing value imputation, and Recursive Feature Elimination (RFE). The second step focuses on model training and hyperparameter optimization: a systematic comparison will be conducted across traditional machine learning models (Random Forest, Logistic Regression, Support Vector Machine [SVM], Naïve Bayes, Gradient Boosting, XGBoost) and deep learning architectures (Long Short-Term Memory [LSTM], Convolutional Neural Network [CNN]). To minimize overfitting and enhance model generalizability, 10-fold cross-validation combined with Bayesian hyperparameter optimization (via the Hyperopt library) will be employed. The third step is model evaluation: craving state will serve as the Primary Outcome Label, and craving severity will act as the Secondary Outcome Label. The Weighted F1-score (to address class imbalance) and the Area Under the Receiver Operating Characteristic Curve (AUC-ROC) (to quantify overall discriminative capacity) will be adopted as core metrics (accuracy will not be prioritized due to potential class imbalance). Finally, model interpretation: SHAP (SHapley Additive exPlanations) values will be computed to interpret the best-performing model, which will quantify the contribution of each input feature (e.g., AU12 intensity, Root Mean Square of Successive Differences [RMSSD]) to craving state prediction.

For Research Objective 4, the focus will be on establishing a treatment efficacy prediction model. This model will be used to assess changes in alcohol craving observed before and after conventional inpatient treatment, and to explore how to construct such a model based on baseline expression characteristics. Six weeks after the completion of inpatient treatment, a repeated measures analysis of craving and Action Unit features (limited to the AUD group) will be conducted. The specific steps are as follows: Firstly, Linear Mixed-Effects Models (LMMs) will be used to analyze changes in facial AU features and subjective craving scores between baseline (pre-treatment) and 6 weeks post-treatment. In this analysis: Time (categorical: baseline/6-week post-treatment) and treatment response (continuous: PACS score change) will serve as independent variables; Changes in AU features and changes in VAS scores will serve as dependent variables; Subject ID will act as a random effect; Age, education level, baseline Hamilton Depression Rating Scale (HAM-D) score, and baseline medication use will function as fixed effects. Secondly, a Treatment Efficacy Prediction Model will be developed. In this model: Baseline facial AU features, baseline PACS scores, and baseline HRV indices (RMSSD) will serve as predictors; Treatment response (defined as PACS score reduction) will act as the outcome label; The machine learning framework mentioned earlier will be adopted for model development.

For Research Objective 5, regarding the prediction of relapse risk, we will verify the predictive value of baseline facial action unit (AU) features for alcohol use disorder (AUD) relapse (6-month follow-up). Baseline facial AU features will serve as the independent variable, while the binary outcome (relapse status at 6 months: 1 = relapsed, 0 = abstinent) and time-to-event outcome (days from discharge to first relapse) will be the dependent variables. We will use the following as our analytical methods: binary logistic regression (for relapse status, adjusted for age, education, and baseline PACS score); Kaplan–Meier survival analysis (to plot relapse-free survival curves by baseline AU tertiles); and Cox proportional hazards regression (to estimate hazard ratios [HR] of baseline AUs for relapse).Additionally, to explore the association between post-6-week AU features and 6-month relapse, the above binary logistic regression and Cox regression analyses will be repeated, with the IV replaced by post-6-week treatment AU features, to investigate whether short-term treatment-induced changes in facial expressions correlate with long-term relapse outcomes.

### 2.6 Data availability

The data that support the findings of this study are available from the corresponding author, upon reasonable request.

## 3 Discussion

This study focuses on the core exploration of craving assessment and treatment efficacy prediction in populations with Alcohol Use Disorder (AUD). By integrating cross-sectional and longitudinal designs (including a 6-month follow-up), it systematically analyzes the differences in facial expressions among AUD patients, long-term abstainers, and healthy individuals when exposed to alcohol cues. Meanwhile, it incorporates the Visual Analog Scale (VAS), psychological scales, and physiological indicators (Heart Rate Variability [HRV], Skin Conductance [SC]), aiming to construct an objective craving assessment system and a treatment efficacy prediction model, as well as to validate the predictive value of baseline characteristics for long-term relapse risk. Ultimately, this study intends to provide evidence-based support for clinical interventions and relapse prevention in AUD.

The primary objective of this study is to identify objective external biomarkers for the psychological craving state in patients with Alcohol Use Disorder (AUD)—specifically, the differences in facial expressions induced by alcohol cues. By comparing facial expression features among AUD patients, long-term abstainers, and healthy individuals, we may identify subtle yet quantifiable differences in how these groups respond to alcohol cues through facial expressions; such differences may reflect the psychological dependence state associated with craving. Meanwhile, by integrating Visual Analog Scale (VAS) scores and facial expression data from AUD patients and healthy individuals, the craving state and severity assessment system constructed using machine learning will initially achieve the transition from subjective reporting to objective quantification, addressing the limitation that current AUD craving assessments rely excessively on self-reported scales (e.g., VAS, Penn Alcohol Craving Scale [PACS]). Building on this foundation, the study will further analyze changes in VAS scores and facial expressions of AUD patients through longitudinal tracking (at admission and after 6 weeks of inpatient treatment). Combined with the results of psychological scales, a treatment efficacy prediction model will be established using machine learning, enabling dynamic assessment of the improvement effect of short-term treatment on craving states. Moreover, the 6-month follow-up extends the research perspective from short-term treatment efficacy to long-term prognosis—by tracking patients’ relapse status during the follow-up period, we will conduct the first attempt to verify whether baseline facial expression features (especially craving-related facial action units [AUs]) can serve as early predictors of relapse. If this hypothesis is validated, it will provide a key tool for early identification and early intervention in clinical practice: for instance, developing individualized relapse prevention plans (e.g., intensive psychological interventions, regular monitoring) in advance for patients with high-risk baseline facial expression features. This holds significant clinical practical value for reducing the relapse rate of AUD. Additionally, considering the prevalence of light drinkers in clinical settings, this study innovatively includes non-drinkers and light drinkers in the control group. Through stratified analysis, it distinguishes the facial expression features corresponding to “appetitive responses (53)” and pathological craving, further enhancing the ecological validity of the study results. This ensures that the identified expression features are truly associated with the pathological craving of AUD, rather than general alcohol-approach behaviors.

To further enhance the objectivity and multidimensionality of craving assessment, this study also integrates facial expression features with physiological indicators such as HRV (Heart Rate Variability) and SC (Skin Conductance). HRV and SC can directly reflect the activity of the autonomic nervous system and emotional arousal level; changes in these indicators are not affected by the subjective control of facial movements, which can effectively address the limitation that facial expressions may be deliberately concealed by patients. This multimodal assessment model combining “facial expressions + physiological indicators” not only aligns with the FDA-NIH biomarker criteria but also will demonstrate synergistic value in longitudinal follow-up, jointly enhancing the reliability of long-term prognosis prediction.

During the implementation of this study, some challenges may be encountered, such as the high difficulty in processing and analyzing facial expression feature data; the large sample size and long experimental duration; and the possible poor cooperation of subjects in the alcohol use disorder group, which may lead to a high dropout rate. We will make every effort to find appropriate solutions to these problems, such as seeking support from psychiatrists within the research team.

Despite the rigorous design of this study, there are several limitations. Firstly, only male subjects will be included, which to some extent limits the generalization of the results to female populations with alcohol use disorder. Secondly, the study will be conducted in a single center, with samples mainly from Henan Province. The local social and cultural background as well as drinking habits may affect the patterns of facial expressions and craving responses, so the applicability of the results in other regions or cultural contexts needs further verification. Thirdly, although facial expressions will be collected under standardized laboratory conditions, the mental state, fatigue level, and current mood of the subjects may still affect their facial expressions, and such measurement errors may reduce the stability of the signals. Fourthly, although the machine learning models will undergo cross-validation and hyperparameter optimization, they will not have been validated with external independent samples, and feature extraction completely relies on a single software algorithm, which may be limited by the detection accuracy and robustness of the algorithm itself. Finally, some psychological scales and subjective ratings (e.g., VAS, PACS) will still rely on self-reports from subjects, which involve certain subjectivity and social desirability bias.

To summarize, through its three-tiered design encompassing “cross-sectional comparison, short-term treatment efficacy tracking, and long-term follow-up,” this study will integrate facial expressions, physiological indicators, and machine learning. It will not only construct an objective assessment system for Alcohol Use Disorder (AUD) craving but also, for the first time, explore the predictive value of baseline characteristics for relapse—thereby providing new directions for the precision intervention of AUD.
